# Distribution Characteristics of Sub-Surface Cracks in Fused Quartz Ground with Different Worn Wheels

**DOI:** 10.3390/ma15072443

**Published:** 2022-03-26

**Authors:** Bingyao Zhao, Yonghao Wang, Ying Yan, Kai Wang, Ping Zhou

**Affiliations:** Key Laboratory for Precision and Non-Traditional Machining Technology of Ministry of Education, Dalian University of Technology, Dalian 116024, China; zby666666@mail.dlut.edu.cn (B.Z.); 201365004@mail.dlut.edu.cn (Y.W.); yanying@dlut.edu.cn (Y.Y.); wangkaizhuzai407@163.com (K.W.)

**Keywords:** fused quartz, grinding, damage detection, crack, XFEM, statistical analysis

## Abstract

The crack distribution characteristics in grinding damage have a significant impact on subsequent polishing efficiency and part strength. Grinding tests were carried out on fused quartz using grinding wheels with different wear states. The results showed that the cracks produced by sharp abrasive grits were mainly near chevron cracks and had no preferred direction. However, the blunt abrasive grits produced near partial cone cracks had a preferred direction. At a depth of 96.7 μm from the surface, the amount of cracks in the range of 50°~90° with an inclination angle between the crack and the cutting direction could reach 88.9%. The statistical results showed that the depth and total length of cracks produced by sharp grits were larger than those produced by blunt grits (the maximum crack depth difference was about 40 μm). Therefore, it was concluded that sharp abrasive grits were not conducive to damage control. The findings of this research enhance our understanding of the formation mechanism of grinding damage.

## 1. Introduction

Fused quartz is widely used in optical observation systems [1] and high-power laser devices [2,3,4] due to its excellent properties, such as good spectral transmittance, small internal stress, and better uniformity [5]. Fused quartz, a hard and brittle material, is usually processed by grinding. Grinding damage, including surface fractures and subsurface cracks greatly affects its subsequent polishing efficiency [6,7] and component strength [8,9,10,11]. Therefore, the characteristics analysis of grinding damage is essential to reveal the grinding mechanism to optimize the grinding process.

Grinding damage of hard and brittle materials has been widely investigated based on the indentation fracture mechanics. Lawn et al. [12] established a median crack depth model under sharp indenter conditions and proposed the specific relationship *pαc*^3/2^ (*p* is the applied normal load per particle, *c* is the crack depth). Marshall [13] verified the accuracy of the model, and obtained the relationship between load and crack depth under spherical indenter conditions as *pαc*^9/8^. However, in the actual grinding process, the initiation and propagation of cracks are much more complicated than the indentation of an ideal indenter due to the influence of the distribution and morphology of abrasive grits.

To realize the effective prediction of subsurface crack depth in grinding, scholars studied the cutting depth of abrasive grits calculated according to kinematic parameters and the relationship between damage depth and roughness established based on the indentation formula. Lambropoulos [14] established the relationship between abrasive grit size (*d*) and damage depth (SSD, 0.3*d*^0.68^ < SSD < 2*d*^0.85^). Li et al. [15] established the relationship between SSD and Rz for BK7 glass based on indentation fracture mechanics and grinding kinematics (SSD is sub-surface damage, Rz is the maximum height of profile). The deviation of the theoretical model prediction increased when the surface roughness was large. The reason for this is that the state of the grinding wheel keeps changing, and the cracks produced during the grinding process are quite different from the indentation cracks. Therefore, it is necessary to further study the grinding subsurface crack depth and its generation mechanism.

Compared with the research on the depth of grinding damage, the morphology of crack damage is relatively less studied. In the studies of single grit scratching or the cutting process, there is a basic consensus regarding the subsurface crack morphology of optical glass under the action of classical indenters. Lawn et al. [16] studied the geometric features of the cracking of soda-lime glass under the scratch of a spherical indenter. They found surface damage in the form of partial cone cracks and attributed this to the frictional force between the indenter and the specimen. Houérou et al. [17] analyzed the shape of the crack beneath the scratch by a sharp indenter on the soda-lime silica glass, which had median cracks, radial cracks (also called chevron cracks), and lateral cracks. Although the scratch process is always used to investigate the material removal mechanism of grinding, it is different from the actual grinding process. In the grinding process, abrasive grits act on the damaged surface; its stress state is quite different from the results of single grit scratching on the intact surface. Only a few papers have studied the crack distribution under actual grinding conditions. Tonnellier et al. observed the depth and morphology of the crack of the subsurface ground by Zerodur and ULE, but the crack directional characteristics were neglected (Figure 3 in ref. [18]) [18]. Zhang et al. [19] found that the direction of cracks produced by grinding was consistent with that of slicing. It was believed that the crack directionality reflected the trajectory of the abrasive, but it has not been further studied. Young et al. [20], in the silicon wafer rotary grinding experiment, observed that the distribution characteristics of subsurface cracks were caused by surface polishing, and chevron cracks and partial cone cracks were also found. They concluded that the crack morphology was related to the abrasive grit morphology without further study.

The results of the literature on grinding brittle materials show that the crack distribution characteristics are strikingly different [11,18,19,20,21,22,23]. By observing the subsurface damage of grinding BK7, Li found that the direction of the crack was random because the direction of the abrasive grits load was random [21]. Solhtalab et al. [22] detected the subsurface crack distribution characteristics of BK7 ground by a cup wheel. In their work, the crack distribution was random and did not have directionality (Figure 6 in Ref. [22]). Suratwala et al. [11] used magneto-rheological finishing (MRF) to polish the surface of the ground-fused silica to observe the damage depth, and they found that the distribution of cracks was random and had no obvious directionality. Li et al. [23] observed similar phenomena in the ground surface of fused silica. From the test results obtained in their study, there were great differences in the surface crack morphology. Few scholars analyzed this issue in-depth and gave reasons for the formation of these cracks.

Detailed damage detection was a prerequisite for the accurate analysis of damage distribution characteristics. Among the damage detection methods, destructive methods were still the main methods in grinding, including taper polishing, MRF polishing, chemical etching, and so on. Young et al. [20] used cross-section angle polishing to study the influence of grinding parameters on the SSD depth of silicon wafer prepared by the rotary grinding method. This method can only detect the crack depth in the local area and cannot reflect the overall situation of crack distribution. Suratwala et al. [11] used the MRF taper polishing to detect the SSD of fused silica after grinding and lapping. The measurement area was large, and the depth of different positions was different, so it was not suitable to analyze the characteristics of crack distribution at a specific depth. Spierings [24] used the HF constant etching rate method to compare the etching rate between the defect surface and the substrate to determine the SSD depth. However, the crack distribution characteristics cannot be observed by this method.

In this paper, the sub-surface crack distribution characteristics of grinding fused quartz were studied. Further research on the sub-surface crack distribution characteristics is very important for understanding the grinding removal mechanism of brittle materials and the evaluation of the strength of the ground surface. Through layer-by-layer polishing and image process techniques, the characteristics of large-area crack distributions at a certain depth of the ground surface were obtained, and the influence of the change in the abrasive grit morphology on the surface of the grinding wheel on the crack morphology was studied. In addition, the extended finite element simulation of the single abrasive scratching of fused quartz was carried out, and the reasons for the formation of large-area crack distribution characteristics were analyzed.

## 2. Experimental Design

### 2.1. Sample Preparation and Grinding Wheel

The fused quartz samples used in these tests were 20 mm × 20 mm × 10 mm in size, the SiO_2_ content reached 99.9% (Shanghai Duopu Optical Materials Co., Ltd., Shanghai, China), and the material properties are listed in Table 1. Before the grinding process, two parallel surfaces were polished to ensure the smoothness and flatness of the sample surface. The surface roughness (Ra) after polishing was about 1 nm, and the surface has no cracks. Two single-layer electroplated diamond wheels (Dongguan Xuqi Hardware Co., Ltd., Dongguan, Guangdong, China) with a size of φ15 × 10 mm and a grit size of #80 were used in the grinding experiment. Grinding wheel A was an undressed wheel, and grinding wheel B was dressed by Inconel 718 (Shenyang Changpu Superhard Precision Tools Co., Ltd., Shenyang, China).

### 2.2. Experimental Setup and Grinding Parameters

**Figure 1:**
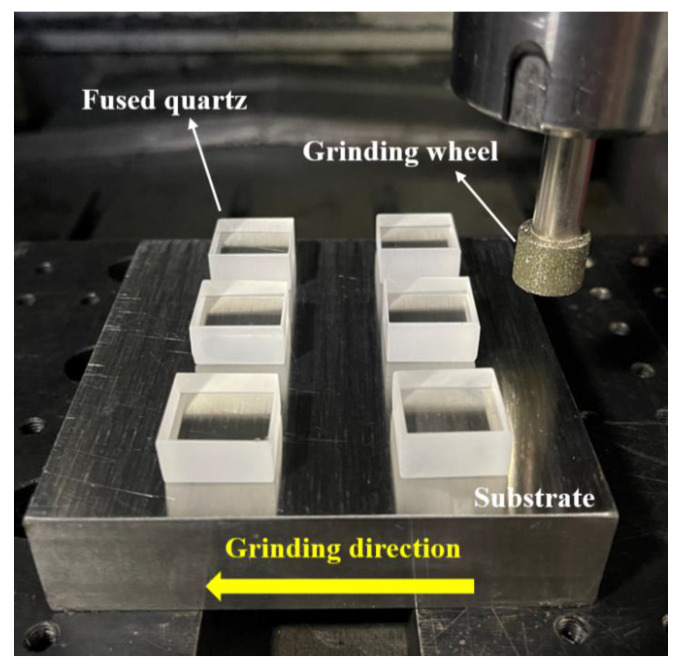
Schematic diagram of the experiment.

The grinding experiment equipment was NHX650 high-speed CNC milling machine (Qinhuangdao Ninghua Machine Tool Co., Ltd., Qinhuangdao, China). As shown in Figure 1, the samples were first bonded to the steel plate with good parallelism by a binder, and then the hole was fixed on the machine tool by a fixture. During the grinding process, the end surface of the grinding wheel moved along the length of the sample. In this direction, the wheel and the sample surface only performed a single action, without repeating the surface processing. Water-soluble oil was used as the grinding fluid. The grinding parameters used in the experiment are shown in Table 2.

### 2.3. Observation of Grinding Wheels’ Profiles

**Figure 2:**
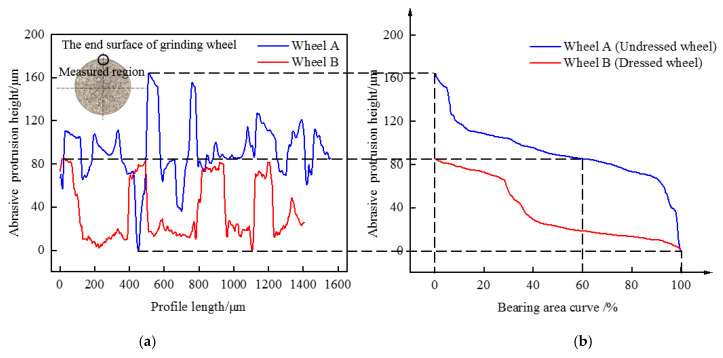
Comparison of abrasive grits morphology of grinding wheel A and B in the peripheral region, the schematic diagram of the end face abrasive grain selection area is shown in (**a**). (**a**) Profiles. (**b**) Bearing area curve based on line profile.

In this paper, fused quartz was mainly removed by abrasive grits on the end face of the grinding wheel. The profile of the peripheral region of the two grinding wheels was observed and measured by laser confocal microscope. The measurement range was 1421 μm × 1065 μm. In Figure 2a, a significant difference in abrasive grit morphology was found between the two wheels. It showed that the abrasive grits of wheel B were flatter compared to wheel A, which was mainly caused by the dressing action on wheel B. Moreover, the protrusion height of wheel B was more consistent due to the long-term dressing, while the height of wheel A had a certain difference, and its overall protrusion height was greater than that of wheel B. Figure 2b shows the bearing area curve (BAC) based on the 3D profile of grinding wheels. It was observed that if the slope at the top of BAC was greater, the shape of the peak region would be sharper and the peaks would be more uneven in height. Thus, the results in Figure 2b also show that the grit protrusion height of grinding wheel B was more consistent and the high peaks were smoother.

### 2.4. SSD Measurement

**Figure 3:**
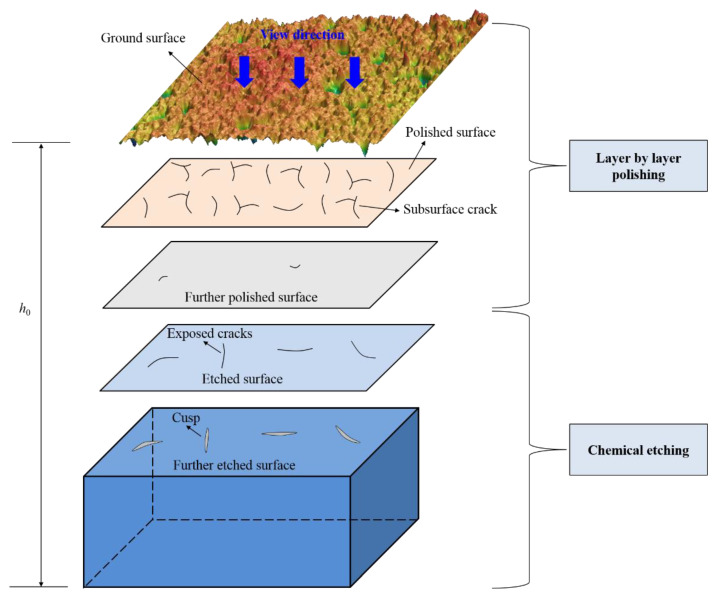
Schematic illustration of layer by layer polishing technology.

To efficiently detect the large-area crack distribution characteristics at a specific depth, a combined method of layer-by-layer polishing and chemical etching is proposed in this paper. The specific steps are shown in Figure 3.

(1) The thickness *h*_0_ of the sample was measured after grinding, then the surface was polished with commercial colloidal silica slurry (COMPOL, FUJIMI Corporation, Japan) under a pressure of 1.8 psi to minimize the possibility of continuous crack propagation. The removal depth at different locations (10 points) of the sample was measured with a spiral micrometer (accuracy of 1 μm);
(2) When the surface fracture zone was completely removed, the surface was corroded (room temperature 20 °C, 5% HF, 15% NH_4_F, Tianjin Damao Chemical Reagent Factory, Tianjin, China, etching time 3 min) at a certain depth of each polishing removal, and the crack morphology was exposed. The crack morphology distribution was tested by a microscope (KEYENCE VK-X260K, Osaka, Japan);
(3) When there were a few isolated cracks left on the surface, the sample was chemically etched to open the crack to form the cusp. Etching conditions were: room temperature 20 °C, 5% HF, 15% NH_4_F, and etching time was 4 min. After etching, ultrasonic cleaning was performed in anhydrous ethanol (Tianjin Dongli District Tianda Chemical Reagent Factory, Tianjin, China) for 20 min;
(4) A laser confocal microscope (KEYENCE VK-X260K, Osaka, Japan, 10× lens, observation range of a single image was 1421 μm × 1065 μm) was used to test the crack morphology after large-scale corrosion. Based on the principle of constant velocity etching, the depth distribution characteristics of the crack tip were obtained.

**Figure 4:**
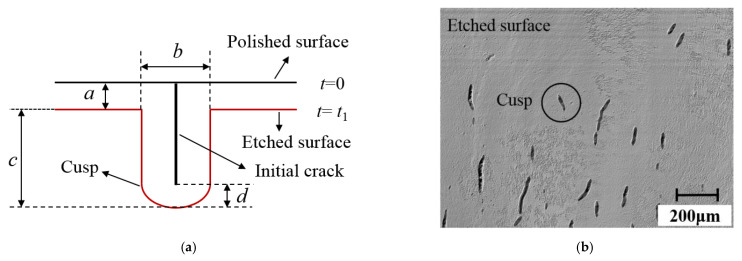
Schematic illustration of chemical etching technique. (**a**) Corrosion model, (**b**) Crack distribution characteristics after corrosion at a subsurface depth of 96.7 μm under the action of the dressed wheel.

At first, the individual crack was closed before etching. Under chemical action, when the rounded cusp was formed, the subsurface cracks were fully expanded. According to Wong’s research [26], the crack width after corrosion was twice that of the etching depth. This proved that the cracks grew isotropically, and the etching process was a constant velocity corrosion so that the depth of SSD could be obtained. The corrosion model was shown in Figure 4a. In this experiment, the crack morphology after etching was shown in Figure 4b; the average width of more than 20 cracks after corrosion was 16.6 μm, while the average corrosion depth was 7.9 μm. The results proved that the etching process met the requirement of constant corrosion. The combined method provided the distribution characteristics of large-area cracks at different depths without introducing new damage, including information, such as crack morphology, length, depth, and other information.

## 3. Finite Element Modeling

The extended finite element method (XFEM) in Abaqus was used to simulate the scratch of a single abrasive grit of fused quartz. The specimen dimension was 1.6 mm × 0.5 mm × 0.3 mm (The selection of the size avoided the influence of the boundary in the simulation). The material (quartz glass) was assumed to be isotropic and exhibited elastic-plastic behavior. The properties of the specimen were Young’s modulus *E* = 70 GPa, Poisson’s ratio *v* = 0.15, and density *ρ* = 2.2 g/cm^3^. The whole model adopted the C3D8R element type, and the completely constrained displacement condition was imposed on the bottom of the specimen model.

**Figure 5:**
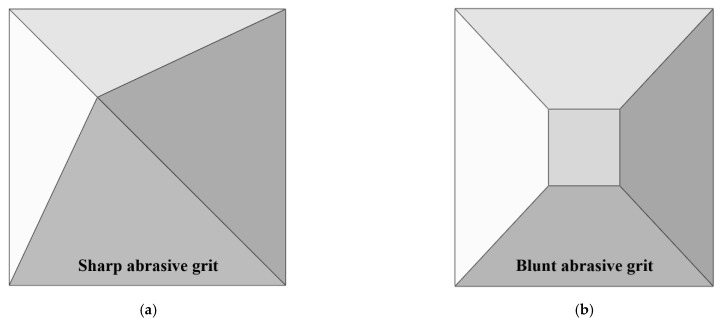
Simulation models of different grits after wear: (**a**) Sharp abrasive grit, (**b**) blunt abrasive grit.

A sharp grit and a blunt grit, as shown in Figure 5, were employed in the simulation to simulate the undressed grinding wheel and the dressed grinding wheel, respectively. Since the stiffness of the grit was much higher than that of the work material, to simplify the calculation, the grit was assumed to be a rigid body.

**Figure 6:**
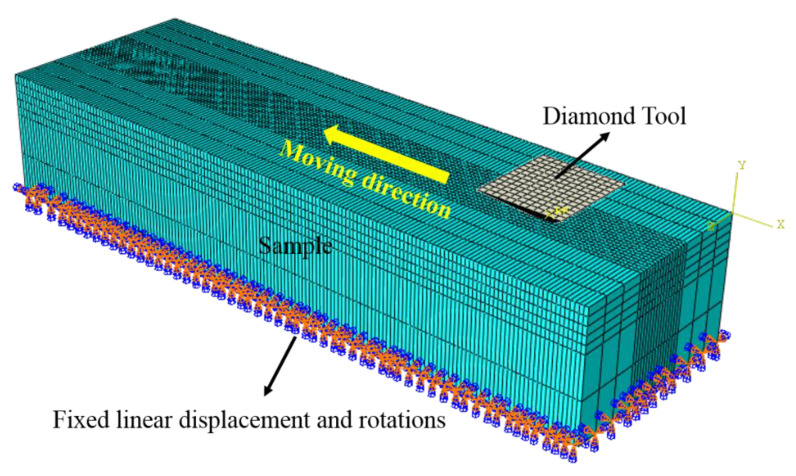
The 3D simulation of single-grit scratching.

The calculations were carried out in two steps: the first step was the grit indentation process (*a*_p_ < 10 μm), and then the scratch movement began along the length direction of the sample after the pressing process was completed (*v* = 5 mm/s). The coefficient of friction was set as 0.2. To ensure the accuracy of the simulation and save calculation time, the model had a refined mesh at the contact area between the grit and the sample. The 3D simulation model of single abrasive scratching was shown in Figure 6.

XFEM was used to simulate the initiations and growths of cracks [27,28,29], and the bilinear traction-separation law was used as material damage in the analysis of XFEM. The description of the traction-separation law was mainly divided into three parts [30,31]. The first part was the damage initiation point corresponding to the maximum principal stress and the critical displacement ($\sigma_{\max}$, $\delta_{c}$). The damage would be initiated when the applied stress reached $\sigma_{\max}$. The second part corresponded to the linear decline part. This part was the degeneration of the stiffness tensor according to the softening branch during further loading, which was the process of damage evolution. The third part corresponds to the final stage of destruction. When δ reached the critical value of $\delta_{c}$, cracks were formed. The maximum principal stress value for controlling fracture was 0.3 GPa. The initial σ − δ law was related to the fracture energy Γ and σ, and the area of the curve σ − δ corresponded to the fracture energy:

(1) $$\Gamma =\frac{(1-v^{2})K_{\mathrm{IC}}^{2}}{E}=\frac{1}{2}\sigma_{\max}\delta_{c}$$

where *E*, *v*, *K*_IC_ and $\delta_{c}$ are the Young’s modulus, Poisson’s ratio, fracture toughness, and the displacement at failure, respectively. Since material softening may cause serious convergence problems, a smaller artificial viscosity coefficient was introduced in the material setting [32].

## 4. Results and Discussion

### 4.1. Crack Morphology and Directionality Generation Mechanism

#### 4.1.1. Crack Distribution Morphology at Different Depths

**Figure 7:**
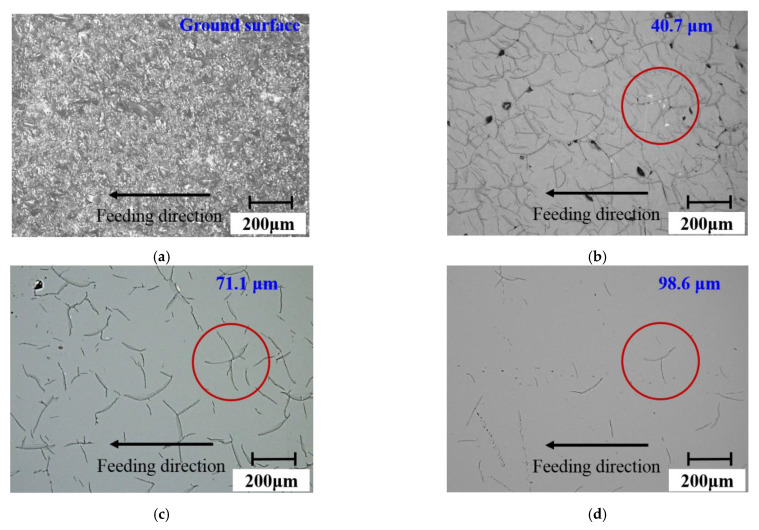

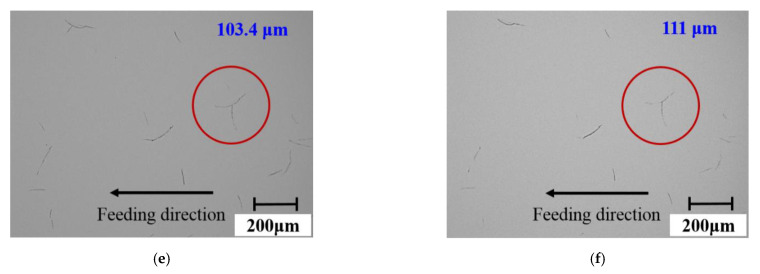
Distribution characteristics of cracks at different subsurface depths under the action of the undressed wheel, subsurface depth (**a**) 0 μm, (**b**) 40.7 μm, (**c**) 71.1 μm, (**d**) 98.6 μm, (**e**) 103.4 μm, (**f**) 111 μm. The red circle positions in the figure show the variation in a bifurcation crack with subsurface depth.

**Figure 8:**
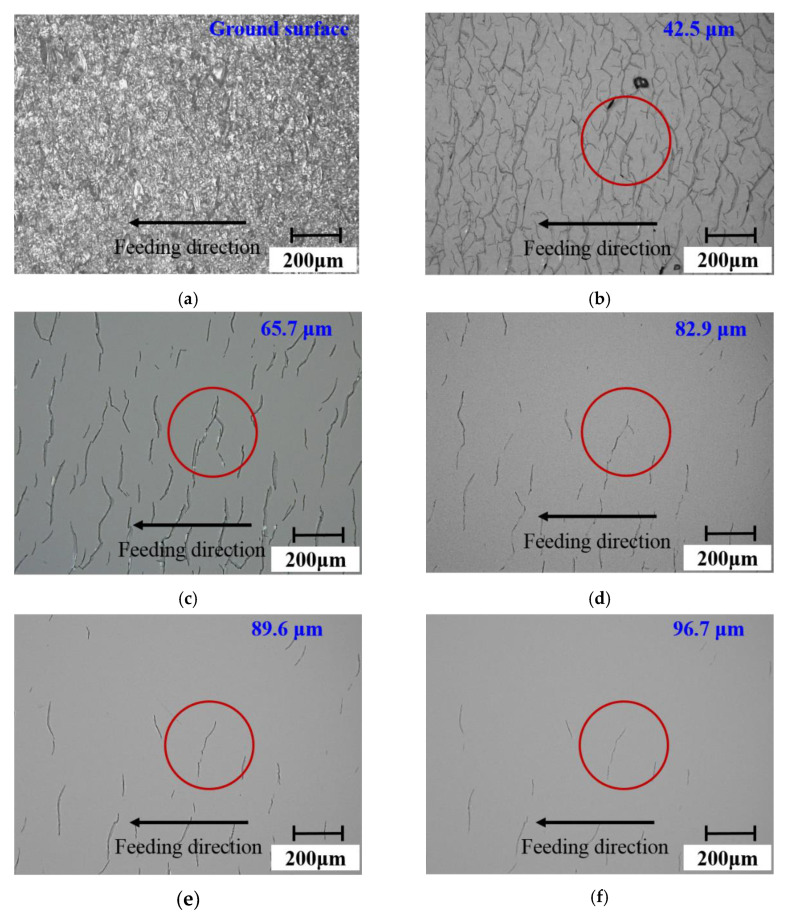
Distribution characteristics of cracks at different subsurface depths under the action of the dressed wheel: subsurface depth (**a**) 0 μm, (**b**) 42.5 μm, (**c**) 65.7 μm, (**d**) 82.9 μm, (**e**) 89.6 μm, and (**f**) 96.7 μm. The red circles in the figure show the variations in a bifurcation crack with subsurface depth.

Figure 7 and Figure 8 show the surface and subsurface damage characteristics of the fused quartz ground by the undressed grinding wheel and the dressed grinding wheel, respectively. From Figure 7a and Figure 8a, it was found that the surface after grinding showed a large number of brittle fractures. With the increase in polishing depth, surface brittle fractures were gradually removed, and the subsurface micro-cracks were exposed. At the shallow position, as shown in Figure 7b,c and Figure 8b,c, the cracks were denser and there were a lot of intersections. These cracks included intersections between different cracks and the bifurcations of the same crack. With the increase in depth, as shown in Figure 7d,f and Figure 8d,f, the number of cracks decreased significantly and the intersections almost disappeared. Only the bifurcations, as exemplified by the solid line section in Figure 7 and Figure 8, were left. At a deeper depth, isolated and clear cracks appeared.

Furthermore, comparing the results of the two grinding wheels, after the fused quartz was ground by the undressed grinding wheel (as shown in Figure 7f), the subsurface cracks were mainly bifurcation cracks. The difference was that most of the subsurface cracks after grinding by the dressed wheel (as shown in Figure 8f) were not bifurcation cracks and had a preferred direction. Additionally, the number of bifurcations crack produced by the dressed wheel was significantly less than that produced by the undressed wheel. According to the literature [33], Misra et al. found that chevron cracks came from sharp grits, while partial cone cracks came from the action of blunt grits in the silicon wafer scratch test. In this study, the crack morphologies produced by the undressed wheel and the dressed wheel were similar to these two kinds of cracks. The chevron crack morphology was a bifurcation crack in the direction of motion. This conclusion is in good agreement with the grinding results.

#### 4.1.2. Factors Affecting Crack Directionality

**Figure 9:**
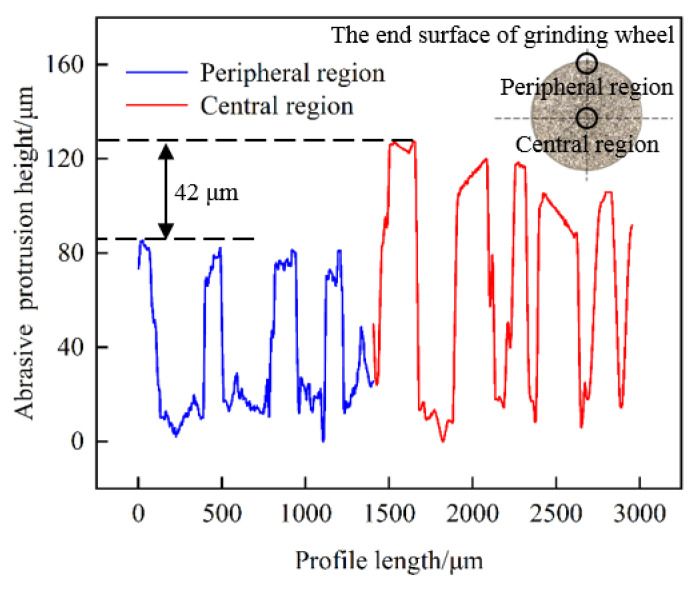
Comparison of abrasive grits’ protrusion height of the dressed grinding wheel in the peripheral region and center region.

**Figure 10:**
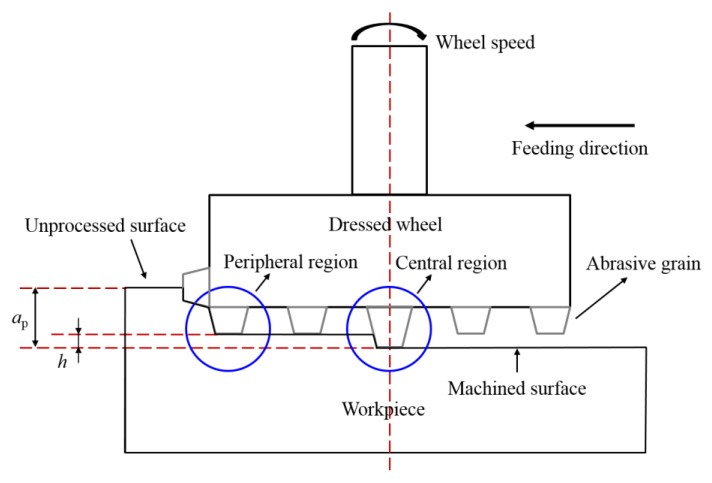
Schematic diagram of abrasive grits on the end face of the dressed grinding wheel.

The direction of cracks produced by single-point scratching was related to the direction of abrasive grit movement [16,17]. During the actual grinding process, the direction of the end face grinding movement was divided into two parts: one was the direction of movement of abrasive grits at a certain moment, and the other was the direction of feed movement. As displayed in Figure 8f, the subsurface crack direction was almost perpendicular to the feeding direction. To further explain crack directionality, it was necessary to clarify the morphology and protrusion height of abrasive grits. The shape of grits directly affected the type of cracks. The protruding height of abrasive grits determined which grits dominated the material removal process. A comparison of the protruding height of abrasive grits in different regions of the dressed wheel was shown in Figure 9. The protruding height of abrasive grits in the peripheral region was significantly smaller than that in the central region (the maximum height difference was approximately 50 μm). This was because the abrasive grits worn in the peripheral region were more serious than those in the central region. Although the abrasive grits at the peripheral region first acted on the surface of the specimen, the protrusion height of the abrasive grits at the central region was higher, which further removed the material and produced deeper cracks, as shown in Figure 10. The cutting direction of the abrasive grits in the central region was consistent with the feeding direction, and thus the crack direction was approximately perpendicular to the feed direction. For the undressed grinding wheel, the grit protruding height was random, and the movement speed was different at different positions; therefore, the direction of the generated cracks was without obvious directionality.

### 4.2. Quantitative Statistics of Crack Distribution Characteristics

#### 4.2.1. Statistical Analysis of Crack Directionality

**Figure 11:**
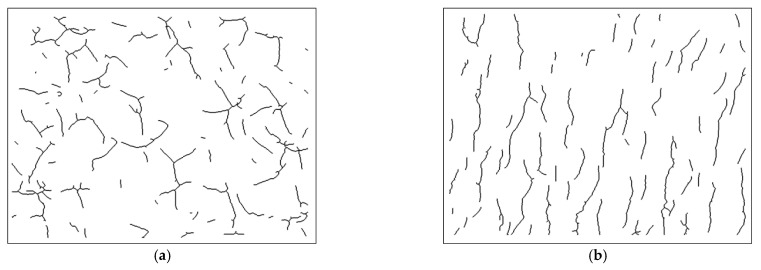
Image processing of subsurface crack morphology under two grinding wheels; the subsurface cracks were black, and the surrounding area was white: (**a**) is the result after processing in Figure 7c, and (**b**) is the result after processing in Figure 8c.

Quantitative statistics of crack morphology were conducted by image processing function in Matlab. The optical photographs were firstly processed in gray scale. After Gaussian filtering and median filtering to remove noise, the edge contours in the images were extracted to find the crack position, and the impurity points, other than the cracks, were removed. Finally, the images were expanded and refined. In Figure 11, the cracks in the processed image are presented as black lines, and the parts without cracks are displayed in white, using Figure 7c and Figure 8c as examples.

**Figure 12:**
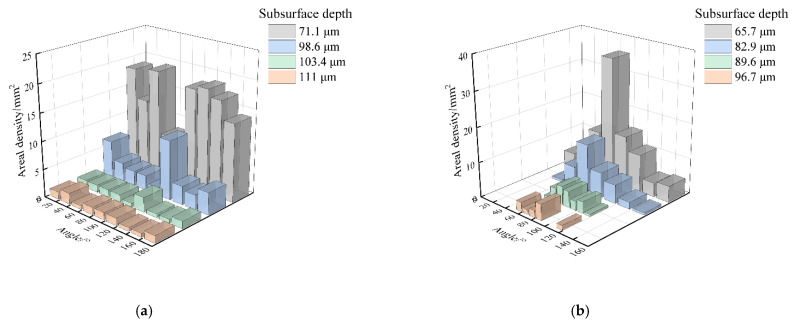
Variation in crack angles with depths under different worn wheels: (**a**) Undressed wheel and (**b**) dressed wheel.

Furthermore, the slope of each crack was obtained by linear fitting and then the inclination angle was calculated and the statistical results were shown in Figure 12. Since the cracks had bifurcations, they need to be calculated separately, and the range was set to 0°–180° to reflect the difference in directionality. It was found that the distribution of crack angles shown in Figure 12a had no preferred direction at any depth. Differently, as shown in Figure 12b, 71.6% of the cracks inclined between 50° and 90°. With the gradual increase in subsurface depth, some shallow cracks were removed and the crack directionality became increasingly clear. Finally, the cracks in the range of 50° to 90° from the cutting direction accounted for 88.9% at a depth of 96.7 μm. Considering the grit morphology and protruding height, the dressed wheel was mainly dominated by grits in the central area of the end face, which produced deep cracks (as shown in Figure 9 and Figure 10). The cutting direction of these grits was approximately the same as the feeding direction. Therefore, most of the cracks produced by these grits had similar angles.

#### 4.2.2. Statistical Analysis of Crack Depth

**Figure 13:**
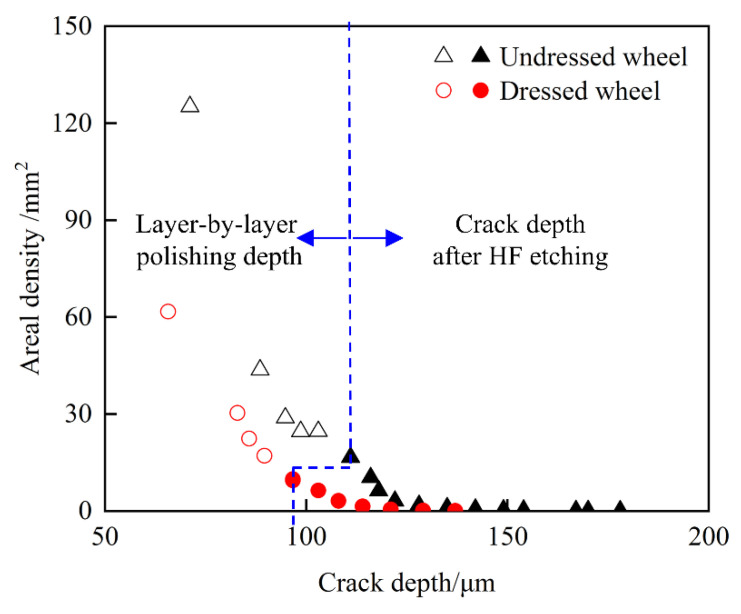
Variation in crack areal density with crack depth under the action of different grinding wheels.

Based on the constant corrosion in Section 2.4, the actual crack depths under the action of two grinding wheels were counted separately. The cracks were not completely perpendicular to the surface in the depth direction. The cracks after corrosion were measured from the back of the sample by a laser confocal microscope to obtain the real crack depth. The measured area was about 18 mm^2^. In this method, the influence of the refractive index of silica quartz on the crack depth was negligible. The layer-by-layer polishing depth and the crack depth after corrosion were combined, and the results are shown in Figure 13. The points where the blue dashed lines crossed included both polishing and corrosion data. The reason that the data points were almost coincident was that the cracks after polishing were divided into multiple cracks. Part of the corroded cracks was expanded and connected, and the other part of the closed cracks appeared by corrosion. The number of cracks per unit area and the depth of cracks produced by the action of the undressed wheel was greater than those produced by the dressed wheel. Notably, the value of a certain data point was greater than that of a previous data point. The reason for this was that, as the polishing depth increases, a crack was divided into several separate cracks, and the number of cracks increased. According to the types of cracks, the cracks can be approximated as radial cracks and Hertzian cracks based on indentation fracture. The load generated by the sharp indenter was less than that of the blunt indenter under the same cutting depth. When the load range was 5–60 N, the radial cracks were more likely to occur compared to Hertzian cracks, and the radial crack depth was greater than the Hertzian depth [11]. The normal load in this paper was consistent with the results of the other researchers, which proved the reliability of the experimental results.

#### 4.2.3. Statistical Analysis of Crack Length

The crack length was calculated by sequentially traversing the rows and columns of the image to determine the point adjacency of the crack region. As the pixel coordinate values of the crack edges changed, the crack length increased by *d*_p_ or $\sqrt{2}d_{p}$, which was then accumulated to obtain the final crack length [34]. With this method, the crack length *l* (pixel) was first calculated and then converted to the actual crack length *L* (mm) by the determined CCD image length correction factor scale (mm/pixel):

(2) L=scale×l

**Figure 14:**
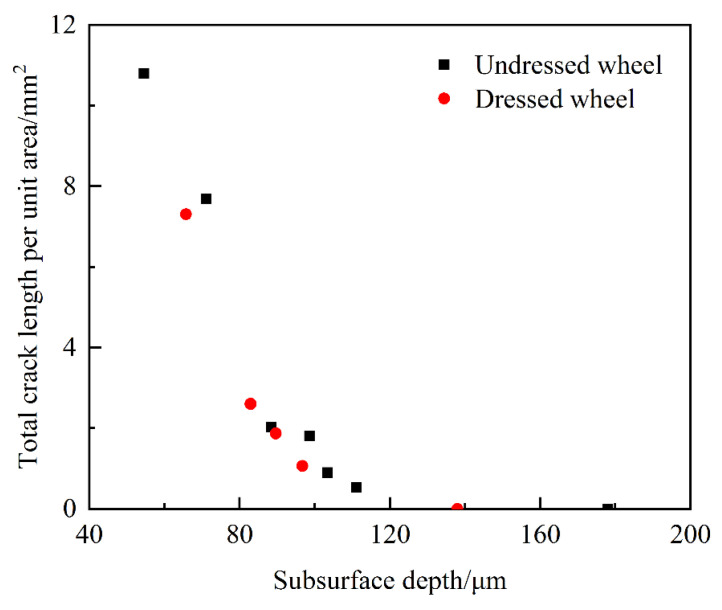
Results of the variation in total crack length per unit area with depth under different grinding wheels.

In this paper, the calibration CCD image length correction factor was 1.38889 mm/dpi. Furthermore, the accuracy of the method was verified by the analytical solution of the parabolic length; the error was 7.1%, which proved the feasibility of this method. The results of the crack length are shown in Figure 14. The length of cracks produced by both grinding wheels decreased with the increasing crack depth. In the deepest crack, after corrosion, the crack length was 0. It was also found that the total crack length per unit area at different depths produced by the undressed wheel was greater than that produced by the dressed wheel.

The method of calculating the length of cracks produced by scratching based on a spherical grit is described in [11].

(3) $$L≅\frac{\pi }{2}{\left(\frac{2}{3}\frac{k}{E}Pd\right)}^{\frac{1}{3}}$$

*L* is the crack length for a spherical grit, *P* is the initiation load, *d* is the ‘effective’ abrasive diameter assuming a spherical grit, and *k* is a ratio of material constants given by:

(4) $$k=\frac{9}{16}\left[\left(1-v^{2}\right)+\left(1-v_{p}^{2}\right)E/E_{p}\right]$$

*v* and *v*_p_ are the Poisson’s ratios for the glass and abrasive grit, respectively. *E* and *E*_p_ are the Young’s moduli of the glass and grit, respectively.

In the actual grinding process, the grinding forces generated by the two grinding wheels were approximately 20 N and 60 N, respectively [35]. From the above theoretical model, the single crack length produced by a spherical grit was about 58 μm. Based on the assumption that the cracks produced under sharp abrasive grains were half-penny cracks, the single crack length and crack depth were approximately equal to 70 μm. In addition, the number of cracks produced by the undressed grinding wheel was greater than that of the dressed grinding wheel under the same measurement area. Therefore, the total crack length per unit area produced by the undressed wheel was greater than that of the dressed wheel.

### 4.3. XFEM Simulation Results

**Figure 15:**
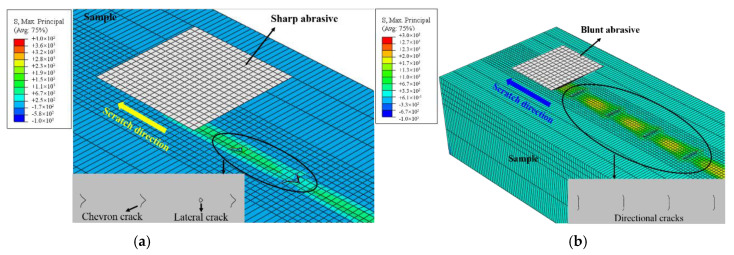
XFEM simulation results of different abrasive grits scratching quartz: (**a**) sharp grit, (**b**) blunt grit. The inserted figures show the enlarged view of the crack results.

Figure 15a shows that sharp grit produced chevron cracks and lateral cracks, and the chevron cracks had a directivity, along with the scratching. Different from the grinding conditions, the scratching only moved in a specific direction. The movement direction of the effective grit was constantly changing, and the grit height was random during the actual grinding process, so the cracks generated by scratching were directional. This result was in agreement with Houérou’s result [17]. Lateral cracks near the surface were removed with a layer-by-layer polishing process, and chevron cracks remained.

Figure 15b showed that blunt-grit-produced cracks, similar to the partial cone and this type of crack, were generated along the scratching direction, which was similar to the shape of the cracks produced by the actual grinding in this paper. This result was somewhat different from the crack morphology produced in the scratch study under a standard indenter [16,36,37] due to the grit shape. The reason for this crack was that the tangential load increased the tensile stress at the trailing edge of the contact zone between the grit and the sample during scratching. It reduced the load required for crack initiation and led to the preferential formation of fracture at the trailing edge of the grit.

Further, a comparison between the normal force results of the two simulation models revealed that the normal force of the sharp grit at an indentation depth of 2 μm was much lower than that of the blunt grit (2.9 N). At this time, no crack was produced under the sharp grit, while the blunt grit produced cracks. When the indentation depth was increased to 5 μm, the normal force reached 0.7 N and chevron cracks were produced. This phenomenon was consistent with grinding tests. Through XFEM simulation, the rationality of the grinding test results was verified.

## 5. Conclusions

This paper investigated the distribution characteristics of sub-surface cracks in fused quartz ground with different worn wheels. The main conclusions of this paper are:

1. Both for the undressed grinding wheel and blunt grinding wheel, the cracks appeared to be cross-connected at the position with small depth. With the increase in the depth, the crack intersection gradually decreased. At a deeper depth, the cracks produced by the sharp abrasive grits of an undressed grinding wheel were mainly near chevron cracks, and the blunt abrasive grits of the dressed grinding wheel were mainly produced near partial cone cracks. Distribution characteristics of cracks at deeper depths were consistent with the ideal single scratch crack;
2. It was found that the cracks produced by the sharp abrasive grits of an undressed grinding wheel had no preferred direction. However, the blunt abrasive grits of the dressed grinding wheel had a preferred direction, and the cracks in the range of 50° to 90° from the cutting direction accounted for 88.9% at a depth of 96.7 μm from the surface. XFEM simulation was used to verify the rationality of the grinding test results;
3. The depth of cracks produced by sharp grits and the total length of cracks per unit area were larger than those produced by blunt grits. This showed that sharp abrasive grits were not conducive to damage control. It can also be inferred that the undressed grinding wheel had a lower strength for grinding fused quartz parts because the fracture strength was determined by the crack depth and density.

The research findings of this paper had practical significance for understanding the formation mechanism of grinding damage under different wheel conditions. Based on the information obtained regarding crack distribution characteristics, it was clear that the dressed wheel could give better results before grinding, which was beneficial for optimizing the grinding process.

Future studies should aim to optimize grinding parameters and establish the relationship between grinding damage depth and strength.

## Figures and Tables

**Table 1:** Material properties of fused quartz [25].

Material	Density *ρ*(g/cm^3^)	Young’s Modulus *E*(GPa)	Poisson’s Ratio *ν*	Vickers Hardness *H*(GPa)	Fracture Toughness *K*_Ic_(MPa·m^1/2^)
Fused quartz	2.2	70	0.15	8.7	0.75

**Table 2:** Grinding parameters.

Grinding Parameter	Values
Depth of cutting, *a*_p_(μm)	100
Grinding wheel speed, *v*_s_(r/min)	5000
Workpiece feed speed, *v*_w_(mm/min)	100

## Data Availability

All data generated or analyzed during this study are included in the present article.

## References

[1] Stepp L.M., Strom S.E. (2004). The Thirty-Meter Telescope project design and development phase. Second Backaskog Workshop on Extremely Large Telescopes.

[2] Campbell J.H., Hawley-Fedder R.A., Stolz C.J., Menapace J.A., Borden M.R., Whitman P.K., Yu J., Runkel M.J., Riley M.O., Feit M.D. (2004). NIF optical materials and fabrication technologies: An overview. Optical Engineering at the Lawrence Livermore National Laboratory II: The National Ignition Facility.

[3] Yu H., Jing F., Wei X., Zheng W., Zhang X., Sui Z., Li M., Hu D., He S., Peng Z. (2008). Status of prototype of SG-III high-power solid-state laser. XVII International Symposium on Gas Flow, Chemical Lasers, and High-Power Lasers.

[4] Néauport J., Ambard C., Bercegol H., Cahuc O., Champreux J.P., Charles J.L., Cormont P., Darbois N., Darnis P., Destribats J. (2008). Optimizing fused silica polishing processes for 351nm high-power laser application. Laser-Induced Damage in Optical Materials.

[5] Englert M., Hartmann P., Reichel S. (2014). Optical Glass: Refractive index change with wavelength and temperature. Optical Modelling and Design III.

[6] Zhou Y., Funkenbusch P.D., Quesnel D.J., Golini D., Lindquist A. (1994). Effect of Etching and Imaging Mode on the Measurement of Subsurface Damage in Microground Optical Glasses. J. Am. Ceram. Soc..

[7] Steele W.A., Miller P.E., Suratwala T.I., Menapace J.A., Wong L.L., Davis P.J. (2005). MRF applications: Measurement of process-dependent subsurface damage in optical materials using the MRF wedge technique. Laser-Induced Damage in Optical Materials.

[8] Miller P.E., Suratwala T., Wong L.L., Feit M.D., Menapace J.A., Davis P.J., Steele R.A. (2005). The distribution of subsurface damage in fused silica. Laser-Induced Damage in Optical Materials.

[9] Cook R.F., Roach D.H. (1986). The effect of lateral crack growth on the strength of contact flaws in brittle materials. J. Mater. Res..

[10] Daphalapurkar N.P., Ramesh K., Graham-Brady L., Molinari J.-F. (2011). Predicting variability in the dynamic failure strength of brittle materials considering pre-existing flaws. J. Mech. Phys. Solids.

[11] Suratwala T., Wong L., Miller P., Feit M., Menapace J., Steele R., Davis P., Walmer D. (2006). Sub-surface mechanical damage distributions during grinding of fused silica. J. Non-Crystalline Solids.

[12] Lawn B.R., Evans A.G., Marshall D.B. (1980). Elastic/Plastic Indentation Damage in Ceramics: The MediadRadial Crack System. J. Am. Ceram. Soc..

[13] Marshall D.B. (2010). Geometrical Effects in Elastic/Plastic Indentation. J. Am. Ceram. Soc..

[14] Lambropoulos J. (2000). From abrasive size to subsurface damage in grinding. Opt. Fab. Test.

[15] Li H.N., Yu T.B., Zhu LDa Wang W.S. (2016). Evaluation of grinding-induced subsurface damage in optical glass BK7. J. Mater. Process. Technol..

[16] Lawn B.R., Wiederhorn S.M., Roberts D.E. (1984). Effect of sliding friction forces on the strength of brittle materials. J. Mater. Sci..

[17] Houérou V.L., Sangleboeuf J.C., Dériano S., Rouxel T., Duisit G. (2003). Surface damage of soda-lime-silica glasses: Indentation scratch behavior. J. Non-Cryst. Solids.

[18] Tonnellier X., Morantz P., Shore P., Baldwin A., Evans R., Walker D.D. (2007). Subsurface damage in precision ground ULE® and Zerodur^®^ surfaces. Opt. Express.

[19] Zhang X., Song X., Sun Y., Du X., Zhang C., Zu C. (2020). Distribution characteristics of subsurface damage induced by different machining methods of fused silica. Optics Ultra Precision Manufacturing and Testing.

[20] Young H.T., Liao H.T., Huang H.Y. (2006). Surface integrity of silicon wafers in ultra precision machining. Int. J. Adv. Manuf. Technol..

[21] Li S., Wang Z., Wu Y. (2008). Relationship between subsurface damage and surface roughness of optical materials in grinding and lapping processes. J. Mater. Process. Technol..

[22] Solhtalab A., Adibi H., Esmaeilzare A., Rezaei S.M. (2019). Cup wheel grinding-induced subsurface damage in optical glass BK7: An experimental, theoretical and numerical investigation. Precis. Eng..

[23] Li Y., Zheng N., Li H., Hou J., Lei X., Chen X., Yuan Z., Guo Z., Wang J., Guo Y. (2011). Morphology and distribution of subsurface damage in optical fused silica parts: Bound-abrasive grinding. Appl. Surf. Sci..

[24] Spierings G. (1993). Wet chemical etching of silicate glasses in hydrofluoric acid based solutions. J. Mater. Sci..

[25] Tonnellier X. (2009). Precision Grinding for Rapid Manufacturing of Large Optics. Ph.D. Thesis.

[26] Wong L., Suratwala T., Feit M.D., Miller P.E., Steele R. (2009). The effect of HF/NH4F etching on the morphology of surface fractures on fused silica. J. Non-Cryst. Solids.

[27] Huang R., Sukumar N., Prévost J.H. (2003). Modeling quasi-static crack growth with the extended finite element method Part II: Numerical applications. Int. J. Solids Struct..

[28] Stolarska M., Chopp D.L., Moo Es N., Belytschko T. (2001). Modelling crack growth by level sets in the extended ÿnite element method. Int. J. Num. Meth. Eng..

[29] Sukumar N., Prévost J.H. (2003). Modeling quasi-static crack growth with the extended finite element method Part I: Computer implementation. Int. J. Solids Struct..

[30] Hyun H.C., Rickhey F., Lee J.H., Kim M., Lee H. (2015). Evaluation of indentation fracture toughness for brittle materials based on the cohesive zone finite element method. Eng. Fract. Mech..

[31] Lee J.H., Gao Y.F., Johanns K.E., Pharr G.M. (2012). Cohesive interface simulations of indentation cracking as a fracture toughness measurement method for brittle materials. Acta Mater..

[32] Gao Y.F., Bower A.F. (2004). A simple technique for avoiding convergence problems in finite element simulations of crack nucleation and growth on cohesive interfaces. Model Simul. Mater. Sci. Eng..

[33] Misra A., Fininie I. (1979). On the scribing and subsequent fracturing of silicon semiconductor wafers. J. Mater. Sci..

[34] Cao Y., Ying B. (2014). Surface crack detection based on CCD image. Mod. Manu. Eng..

[35] Wang Y., Zhou P., Pan Y., Yan Y., Guo D. (2022). Wheel wear-related instability in grinding of quartz glass. Int. J. Adv. Manuf. Technol..

[36] Lee K., Marimuthu K.P., Kim C.L., Lee H. (2018). Scratch-tip-size effect and change of friction coefficient in nano/micro scratch tests using XFEM. Tribol. Int..

[37] Lofaj F., Németh D. (2018). FEM of cracking during nanoindentation and scratch testing in the hard W-C coating/steel substrate system. Key Eng. Mater..

